# A Closer Look on the Polyhydroxybutyrate- (PHB-) Negative Phenotype of *Ralstonia eutropha* PHB^-^4

**DOI:** 10.1371/journal.pone.0095907

**Published:** 2014-05-02

**Authors:** Matthias Raberg, Birgit Voigt, Michael Hecker, Alexander Steinbüchel

**Affiliations:** 1 Institut für Molekulare Mikrobiologie und Biotechnologie, Westfälische Wilhelms-Universität, Münster, Germany; 2 Department of Environmental Sciences, King Abdulaziz University, Jeddah, Saudi Arabia; 3 Institut für Mikrobiologie, Ernst-Moritz-Arndt Universität, Greifswald, Germany; University Paris South, France

## Abstract

The undefined poly(3-hydroxybutyrate)- (PHB-) negative mutant *R. eutropha* PHB^-^4 was generated in 1970 by 1-nitroso-3-nitro-1-methylguanidine (NMG) treatment. Although being scientific relevant, its genotype remained unknown since its isolation except a recent first investigation. In this study, the mutation causing the PHA-negative phenotype of *R. eutropha* PHB^-^4 was confirmed independently: sequence analysis of the *phaCAB* operon identified a G320A mutation in *phaC* yielding a stop codon, leading to a massively truncated PhaC protein of 106 amino acids (AS) in *R. eutropha* PHB^-^4 instead of 589 AS in the wild type. No other mutations were observed within the *phaCAB* operon. As further mutations probably occurred in the genome of mutant PHB^-^4 potentially causing secondary effects on the cells' metabolism, the main focus of the study was to perform a 2D PAGE-based proteome analysis in order to identify differences in the proteomes of the wild type and mutant PHB^-^4. A total of 20 differentially expressed proteins were identified which provide valuable insights in the metabolomic changes of mutant PHB^-^4. Besides excretion of pyruvate, mutant PHB^-^4 encounters the accumulation of intermediates such as pyruvate and acetyl-CoA by enhanced expression of the observed protein species: (i) ThiJ supports biosynthesis of cofactor TPP and thereby reinforces the 2-oxoacid dehydrogenase complexes as PDHC, ADHC and OGDHC in order to convert pyruvate at a higher rate and the (ii) 3-isopropylmalate dehydrogenase LeuB3 apparently directs pyruvate to synthesis of several amino acids. Different (iii) acylCoA-transferases enable transfer reactions between organic acid intermediates, and (iv) citrate lyase CitE4 regenerates oxaloacetate from citrate for conversion with acetyl-CoA in the TCC in an anaplerotic reaction. Substantial amounts of reduction equivalents generated in the TCC are countered by (v) synthesis of more ubiquinones due to enhanced synthesis of MenG2 and MenG3, thereby improving the respiratory chain which accepts electrons from NADH and succinate.

## Introduction

*Ralstonia eutropha* H16 is a Gram-negative, rod-shaped and facultative chemolithoautotrophic hydrogen-oxidizing bacterium belonging to the β-proteobacteria. *R. eutropha* appears as a ubiquitous inhabitant of soil and freshwater habitats. Although respiration plays the major role in energy generation, this bacterium is well adapted to transient anoxia [1]. *R. eutropha* is able to oxidize molecular H_2_ and various organic compounds. Two hydrogenases catalyze the oxidation of H_2_ during lithoautotrophic growth: one membrane bound hydrogenase transfers electrons into the electron transport chain, and one cytosolic hydrogenase generates reducing power (NADH) for CO_2_ fixation. Autotrophic CO_2_ fixation is mediated by the Calvin-Benson-Bassham cycle [2]. Furthermore, *R. eutropha* is in addition able to use various organic carbon and energy sources for heterotrophic growth including TCA cycle intermediates, sugar acids like gluconic acid, fatty acids or other acids and amino acids. Notably, the capability of H16 to metabolize sugars is restricted to fructose and *N*-acetylglucosamine [3].

Besides playing a key role in investigating hydrogen-based chemolitoautotrophy, *R. eutropha* serves as model organism of polyhydroxyalkanoate (PHA) metabolism since more than 50 years [4], [5]. PHAs are accumulated by a large number of prokaryotes and serve as intracellular storage compounds for carbon and energy and can be used for various applications in industry and medicine due to their thermoplastic properties and biodegradability [6]. The most frequently found polymer of this class is poly(3-hydroxybutyrate) (PHB), which was firstly described by Lemoigne [7] in *Bacillus megaterium*.

PHAs are synthesized in batch or fed-batch cultures under unbalanced growth conditions if a carbon source is present in excess and if another macroelement (N, O, P, S) is depleted at the same time [8], [9]. PHAs may represent the major cell constituent contributing up to 90% of the cell dry weight [10]. Although *R. eutropha* H16 is able to synthesize different PHAs of short carbon chain length [11], PHB is usually the predominant PHA in this bacterium [12], [13].

In *R. eutropha* synthesis of PHB proceeds in three steps catalyzed by the enzymes β-ketothiolase (PhaA), acetoacetyl-CoA reductase (PhaB) and PHA synthase (PhaC) (14–17). The genes for these three enzymes are located in the PHA-operon *phaCAB* [18]–[20]. Whereas PhaC is essential for PHA biosynthesis in *R. eutropha* H16, PhaA and PhaB can be replaced by isoenzymes [21], [22]. A second PHA synthase gene was identified within the *R. eutropha* H16 genome sequence project [23], which putatively encodes an additional PHA synthase in this bacterium. However, this *phaC2* annotated gene is obviously not transcribed in H16 [24]. In the first step, two molecules acetyl-CoA are condensed to acetoacetyl-CoA by a β-ketothiolase (PhaA) [14], [17]. The second reaction is catalyzed by the NADPH-dependent acetoacetyl-CoA reductase (PhaB) yielding (*R*-)3-hydroxybutyryl-CoA (3HB-CoA) [15]. The PHA synthase (PhaC) finally polymerizes the 3HB moieties of 3HB-CoA to PHB [16], [25].

The PHA synthase (PhaC) is regarded as the key enzyme of PHA synthesis and many *phaC* genes have been cloned and characterized [26]–[28] According to their molecular constitution and their substrate specificity PHA synthases are divided into four classes: Class I enzymes form short-chain-length (SCL) PHAs utilizing monomers with three to five carbon atoms (C_3_–C_5_). They are typically found in α- and β-proteobacteria like *R. eutropha* [19], *Rhodococcus ruber* [29], *Methylobacterium extorquens* [30] and *Burkholderia cepacia* [31]. Class II synthases appear in pseudomonads of the rRNA homology group I and accept monomers of medium-chain-length (MCL) comprising six to fourteen carbon atoms (C_6_–C_14_) [28], [32]. In contrast to the afore mentioned enzymes, synthases of class III consist of two different subunits (PhaC + PhaE) and convert short-chain-length (SCL) monomers; they are found in purple sulfur bacteria, sulfate-reducing bacteria and others [33]. Likewise composed of two different subunits (PhaC + PhaR), PHA synthases of class IV are represented by enzymes of *Bacillus sp*. and prefer short-chain-length (SCL) monomers [34].

PHA synthases exhibit generally a broad substrate specificity, and also PhaC of *R. eutropha* catalyzes the synthesis of a large number of PHAs composed of diverse SCL hydroxyalkanoic acids as well as polythioesters (PTEs) consisting of mercaptoalkanoic acids [35], [5].

At the beginning of the 1960ties *R. eutropha* and derived mutants attracted interest as producer of single cell protein (SCP) for animal nutrition from CO_2_ and H_2_ [36]. Similar attempts were conducted with diverse other microorganisms applying various carbon sources [37], but this program was finally stopped due to economic reasons as SCP was not able to compete with classical feedstock and because cells harboured too high concentrations of disturbing RNA. During these investigations, PHB-negative mutants were isolated [38], [39] which became important in research, later. Originally these mutants were isolated because the animals' digestive tracts are unable to degrade PHB which has therefore no nutritional value. The most prominent representative of the PHB-negative mutants became *R. eutropha* PHB^-^4. This strain and similar mutants derived from 1-nitroso-3-nitro-1-methylguanidine (NMG) treatment and are therefore undefined [38]. While several of these mutants showed only reduced PHB synthesis, *R. eutropha* PHB^-^4 did not produce any PHB but showed similar growth as the wild type *R. eutropha* H16. Subsequently, mutant PHB^-^4 became quite famous as it was frequently applied to identify PHB synthase genes from other bacteria. To do so, genomic libraries were screened by phenotypic complementation of mutant PHB^-^4 for restauration of PHB synthesis (reviewed in [27]).

However, although *R. eutropha* PHB^-^4 was applied in a multitude of research projects, a first explanation of the PHB-negative phenotype of his mutant was given only recently when a single nonsense mutation was identified in the *phaC* gene [40]. For a more detailed view on mutant *R. eutropha* PHB^-^4, this study was initiated. The first experimental steps aimed at confirming the mutation in the *phaCAB* operon also in our clone in an independent approach. However, the major goal was to conduct a proteome analysis in order to identify differences in the proteomes of the wild type and mutant PHB^-^4 of *R. eutropha* which may appear due to secondary effects or further mutations in the genome of this undefined mutant.

## Materials and Methods

### Bacterial strains, media and growth conditions

The wild type *Ralstonia eutropha* H16 (DSM 428, SK-No.: 5214) and the 1-nitroso-3-nitro-1-methylguanidine- (NMG-) induced PHB-negative mutant *R. eutropha* PHB^-^4 ([38], DSM 541, SK-No.: 7387) were used in this study. Cultivations in liquid media were done in Erlenmeyer or Klett flasks with baffles on a rotary shaker at an agitation of 125 r.p.m. Cells of *R. eutropha* for proteomic studies were grown in 2 l Klett flasks equipped with baffles at 30°C in 300 ml mineral salts medium (MSM) [41]. These cultivations were done in triplicate to allow generation of 2D PAGE gel replicates from three independent experiments for a subsequent computer based proteome analysis. Media contained sodium gluconate (1.0%, wt/vol) as carbon source and low amounts of NH_4_Cl (0.05%, wt/vol) as nitrogen source to allow PHB synthesis in the wild type H16. When cells had reached the exponential growth phase after 10 hours of cultivation, the first sample was withdrawn, and after in total 26 hours of cultivation, the second sample was withdrawn in the stationary growth phase. These samples of the wild type H16 and mutant PHB^-^4 were subsequently subjected to proteome analyses.

*Escherichia coli* Top10 (Invitrogen) was cultivated in Luria-Bertani (LB) medium [42] at 37°C. Solid media contained 1.8% (wt/vol) agar. If required, 75 µg Ampiciline (Ap) ml^−1^ was added for *E. coli*.

### Isolation, manipulation and transfer of DNA

Isolation of genomic DNA of *R. eutropha* was done according to Marmur [43]. Plasmid DNA was isolated by the method of Birnboim and Doly [44]. DNA manipulations and other standard molecular biology techniques were performed according to Sambrook et al. [42]. Amplifications of genomic DNA were made using Herculase II Fusion DNA polymerase (Agilent Technologies) in an Omnigene HBTR3CM DNA thermocycler (Hybaid) employing oligonucleotide primers mentioned below. Obtained sequences were analyzed using Chromas software (version 1.45, Technelysium Pty Ltd), Genamics Expression software (version 1.100 [http://genamics.com/expression/index.htm]), BLAST online service available on NCBI (National Center for Biotechnology Information [http://blast.ncbi.nlm.nih.gov/Blast.cgi]), and BioEdit [45]. Competent cells of *E. coli* were prepared and transformed by the CaCl_2_ procedure as described by Hanahan [46].

### Sequence analysis of the *phaCAB* operon of the PHB-negative mutant *R. eutropha* PHB^-^4

For sequencing the genomic region of *R. eutropha* PHB^-^4 from 886 bp upstream of gene *phaC* to 123 bp downstream of gene *phaB* comprising the whole *phaCAB* operon, a 4860 bp DNA fragment was generated by PCR using Herculase II Fusion DNA Polymerase (Agilent Technologies) and primers *phaCAB*_fw: GCCGATGAACAGGTCGCGGTTGCC and *phaCAB*_rv: GCCTTGACGGCCCGCGAAACGG applying genomic DNA of mutant *R. eutropha* PHB^-^4 as template. The purified PCR product (peqGOLD Gel Extraction Kit, peqlab) was ligated with vector pJET1.2/blunt and applied for transformation of competent *E. coli* TOP10 cells according to the CloneJET PCR Cloning Kit (Thermo Scientific). Recombinant plasmids were isolated from positive *E. coli* clones, and three independently obtained plasmids were subjected to DNA sequence analysis using the sequencing primers listed in table 1 in order to generate overlapping sequences to ensure sufficient coverage.

**Table 1:** Oligonucleotide primers used for sequencing of the *phaCAB* operon from *R. eutropha* PHB^-^4.

Primer	Description	Location
Seq1_fw	GCAAGCATAGCGCATGGCGTCTCC	Upstream *phaC*
Seq2_rv	GGAGACGCCATGCGCTATGCTTGC	Upstream *phaC*
Seq3_rv	CCCAAAGCGGGAGGGTCTGCC	Upstream *phaC*
Seq4_fw	CCTTGACCGAGCTGGCCGATGCC	Within *phaC*
Seq5_fw	CCAAGACCCGCCAGCGCATCC	Within *phaC*
Seq6_fw	CCAGCTGTTGCAGTACAAGCCGC	Within *phaC*
Seq7_fw	CGCTGCTGACCACGCTGCTGG	Within *phaC*
Seq8_fw	CCGCATGGCTGGCCGGGC	Within *phaC*
Seq9_fw	CCGGAGCAGGTGAGCGAAGTCATCATGG	Within *phaA*
Seq10_ fw	GGCCTGTGGGACGTGTACAACCAGTACC	Within *phaA*
Seq11_fw	GCTCGCAGAACAAGGCCGAAGC	Within *phaA*
Seq12_fw	GGTGGTGATGTCGGCGGCCAAGG	Within *phaA*
Seq13_ fw	GGCATGGGTGGTATCGGAACCG	Within *phaB*
Seq14_fw	GCTGACTGGGACTCGACCAAGACCG	Within *phaB*

DNA sequencing was carried out at the Sequence Laboratories Göttingen (Seqlab). Obtained sequences were assembled and analysed using Chromas software (version 1.45, Technelysium Pty Ltd). Sequence comparisons and alignments were performed using the BLAST online service available on NCBI (National Center for Biotechnology Information [http://blast.ncbi.nlm.nih.gov/Blast.cgi]), BioEdit [45] and ClustalW [47].

### Preparation of protein samples for proteome analysis

To obtain crude extracts from cells which were cultivated in MSM, cells were harvested by centrifugation (15 min, 3,500 *g*, 4°C), and the resulting cell pellets were then treated with crack solution (8 M Urea, 2%, vol/vol, Triton X-114). The volume of crack solution corresponded to the volume of the centrifuged cell suspension, i.e. a pellet that resulted from 50 ml culture broth was resuspended in 50 ml crack solution. The mixture was then incubated for 1.5 h at room temperature on a gyratory shaker to break the cells. PHB and cell debris were removed from the crude extract by centrifugation (1 h, 60,000 to 70,000 *g*, 4°C). Proteins were subsequently extracted from the supernatant by phenol extraction as described before [48]. After aceton precipitation, the washed protein pellet was air-dried by incubation at room temperature to evaporate the acetone, and was then stored at −20°C.

### Rehydration of proteins

An adequate volume of rehydration buffer A (Urea 9 M, CHAPS 4%, wt/vol, DTT 100 mM, H_2_O_dest_. ad 10 ml) in dependency on pellet size was added to the dry protein pellets, and the mixture was then incubated at room temperature for 2 h. To ensure effective rehydration, the samples were stirred several times during this period. Protein solutions were then transferred to 1.5 ml plastic tubes and centrifuged (5 min, 11,000 *g*). The protein concentration in the supernatants was subsequently measured.

### Determination of protein concentration

To reduce the disturbing influence of DTT and Urea [49], which are part of rehydration buffer A, only 5 µl of highly concentrated protein solution were mixed with a volume of 5 ml of Bradford reagent (70 mg Serva Blue G, 50 ml 96%, vol/vol, ethanol, 100 mg 85%, vol/vol, phosphoric acid, H_2_O_dest_. ad 1,000 ml) according to Bradford [50]. After 10 min incubation in the absence of light, the absorption at 595 nm against the reagent blank value was measured. A calibration was done with bovine serum albumin (BSA) in the range from 1 to 1,000 µg.

### 2D PAGE: first-dimension isoelectric focusing (IEF)

An aliquot of protein solution that contained 1.5 mg protein was filled up to 200 µl with rehydration buffer A and was mixed with 150 µl rehydration buffer B (2.5 ml rehydration buffer A, 125 µl ampholyt solution pH 3–10 [Serva], 125 µl Triton X-100, trace amount of Bromophenol Blue). The IEF strips (pH 5–8, 11 cm, BioRad) were passively rehydrated over night at room temperature with 350 µl of prepared protein solutions while overlaid with mineral oil. After rehydration of the strips with the protein solutions, strips were focussed in a focussing tray (while overlaid with mineral oil) by a series of voltage increases: 250 V (1 h), 500 V (1 h), 1,000 V (1 h), 6,000 V (18 h) and 500 V (up to 99 h) at 20°C to a total value of at least 100 kVh.

### 2D PAGE: second dimension and gel staining

The equilibration of IEF strips, 2D gel-run in a DODECA-cell (BioRad) as well as staining and destaining procedures were done exactly as described in Raberg et al. [48].

### Software based analysis of 2D gel images

Images from 2D PAGE were analysed separately for three independent biological experiments using the DECODON Delta 2D software (Decodon GmbH, Greifswald) due to method immanent variations in 2D PAGE gels based on different cultivations, and one representative analysis is presented here in detail. From replicates comprising various samples of identical stages of cultivation (four different modes: (a) exponential growth phase of the wild type *R. eutropha* H16, (b) stationary growth phase of the wild type *R. eutropha* H16 and (c) exponential or (d) stationary growth phase of the PHB-negative mutant *R. eutropha* PHB^-^4; at least three replicates for each sample), average fusion images were generated for each group after the necessary warping steps were performed. Spots were colour coded according to their expression profile in the dual channel images of these fusions.

For further comparison of the protein patterns during the different stages, spot quantities were likewise densitometrically determined by the Delta 2D software. For this, a proteome map comprising all gel images of each of the four replicate groups (representing modes a, b, c and d mentioned above) was created using the union fusion approach of the software. Spot boundaries on the proteome map were detected, transferred to the original images, and spots were automatically quantified by the software. The given spot quantities represent the relative portion (% volume) of an individual spot of the total protein present on the respective average fusion image. Using ANOVA (analysis of variance), differentially expressed proteins between replicate groups were identified. By setting a negative filter (0.75–1.25), only those protein spots were chosen for MALDI-TOF analysis, whose expression increased or decreased by at least 25 percent, to ensure significance.

### Protein preparation, mass spectrometry and data analysis

Spots were cut from 2D gels and transferred to 1.5 ml plastic tubes. MALDI-TOF analysis was performed employing the method of Shevchenko et al. [51] at the ‘Institut für Mikrobiologie, Ernst-Moritz-Arndt Universität, Greifswald, Germany’. For this, proteins were tryptically digested, and the mass spectra of the protein fragments were revealed by MALDI-TOF (Proteome Analyzer 4800, Applied Biosystems, Foster City, CA, USA). The parameters for the measurements were set as described in Voigt et al. [52], except that the signal to noise ratio for the TOF-TOF measurements was raised to 10. These spectra were compared with hypothetical spectra of the *R. eutropha* H16 databank, and proteins were identified by using the computer software “GPMAW” (Lighthouse Data, Denmark) as well as “Mascot”, a programme associated with MALDI-TOF (search parameters as in [52]).

## Results

### Sequence analysis of the *phaCAB* operon of the PHB-negative mutant *R. eutropha* PHB^-^4

The PHB-negative mutant *R. eutropha* PHB^-^4 [38] served in a multitude of research projects, as mentioned before. Beside an initial investigation, which identified a point mutation in the PHB synthase gene *phaC* [40], no further attempts were made to elucidate the PHB-negative phenotype of this NMG-induced and therefore undefined mutant in more detail.

**Figure 1:**
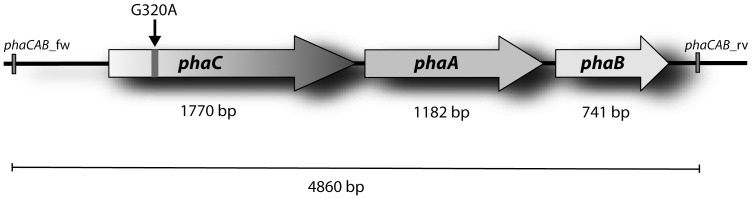
Schematic presentation of the *phaCAB* operon of *R. eutropha* PHB^-^4. Primers *phaCAB_fw* and *phaCAB_rv* were applied to generate the 4,860-bp DNA fragment comprising the *phaCAB* operon. The PCR fragment was subcloned into vector pJET1.2/blunt, and subsequently fragments of three independent hybrid plasmids were sequenced. The arrow marks the observed G320A mutation in *phaC* causing a stop codon obviously leading to a truncated and non functional PHA synthase (PhaC) in strain PHB^-^4.

As phenotypic complementation of strain *R. eutropha* PHB^-^4 to restore PHA synthesis is possible without much effort by expressing an active PHA synthase, a mutation of the *phaC1* gene encoding the only active PHA synthase in *R. eutropha* seemed likely. The second gene, which putatively encodes an additional PHA synthase in the genome of *R. eutropha* H16, *phaC2*, is not transcribed in H16 [24]. Therefore, the data suggesting a nonsense mutation in the *phaC* gene of PHB^-^4 appear convincing [40]. However, before starting the proteome analysis, we wanted to confirm these findings by an independent analysis of the *phaCAB* operon in the mutant clone PHB^-^4 kept for decades in our laboratory. To do so, an about 4.9-kbp DNA fragment comprising the whole *phaCAB* operon was amplified by PCR and cloned into the pJET1.2/blunt vector, as sequencing based on a vector gives much better results and longer reads than from a PCR product. Although a proofreading *Pfu* DNA polymerase was applied in PCR, three independent hybrid plasmids from different *E. coli* clones were analysed, to exclude polymerase caused sequence errors. The sequences obtained fom these three fragments were identical. In contrast, an alignment of the determined sequence of *R. eutropha* PHB^-^4 with the genomic sequence of the wild type H16 identified a point mutation in the *phaC* gene of strain PHB^-^4 (Fig. 1). This observed G320A mutation has a fatal impact as the corresponding base tripplett TGA constitutes a stop codon instead of TGG coding for tryptophan in the wild type. This exchange of one single nucleotide obviously leads to a massively truncated PhaC protein of only 106 amino acids (aa) in *R. eutropha* PHB^-^4 in contrast to 589 aa of the intact protein in the wild type. No other mutations besides this G320A mutation were observed within the *phaCAB* gene cluster of *R. eutropha* PHB^-^4 and the additionally investigated 886 bp upstream region.

### Proteome analysis of the PHB-negative mutant *R. eutropha* PHB^-^4 in comparison to the wild type *R. eutropha* H16

As *R. eutropha* PHB^-^4 is an undefined chemically induced mutant, further mutations in addition to the identified G320A mutation in *phaC* probably occurred in the genome of strain PHB^-^4 which are not necessarily related to the PHB-negative phenotype. On the other side, many mutations can cause secondary effects on a cells' metabolism. To address such unpredictable effects, a 2D PAGE based proteome analysis was conducted in order to identify differences in the proteomes of the wild type H16 and the mutant PHB^-^4 in the exponential and in the stationary growth phase, respectively.

**Figure 2:**
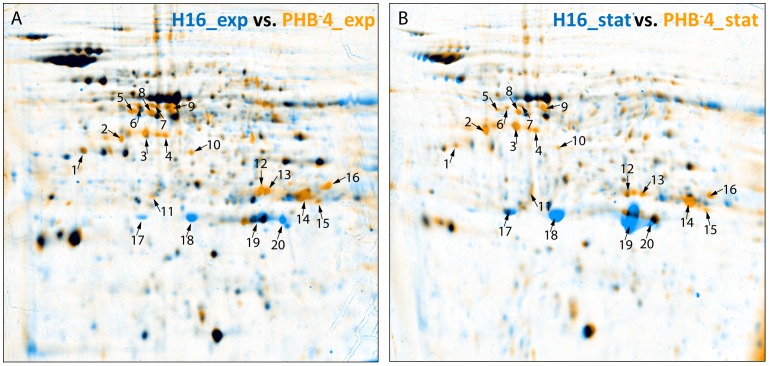
Changes in the proteomes of the PHA-negative mutant *R. eutropha* PHB^-^4 compared to the wild type *R. eutropha* H16. Cells were cultivated in MSM medium under conditions promoting PHB synthesis. Extracted proteins were focused using pH 5 to 8 nonlinear strips. Dual channel images were generated by employing the Delta2D software. (A) Cells of the exponential growth phase. Wild type H16, blue spots; mutant PHB^-^4, orange spots. (B) Cells of the stationary growth phase. Wild type H16, blue spots; mutant PHB^-^4, orange spots. Arrows and numbers mark proteins with significantly different levels of expression in comparison to those of wild type H16 and mutant PHB^-^4. Detailed information about the detected proteins is compiled in Table 2 and Fig. 3.

**Figure 3:**
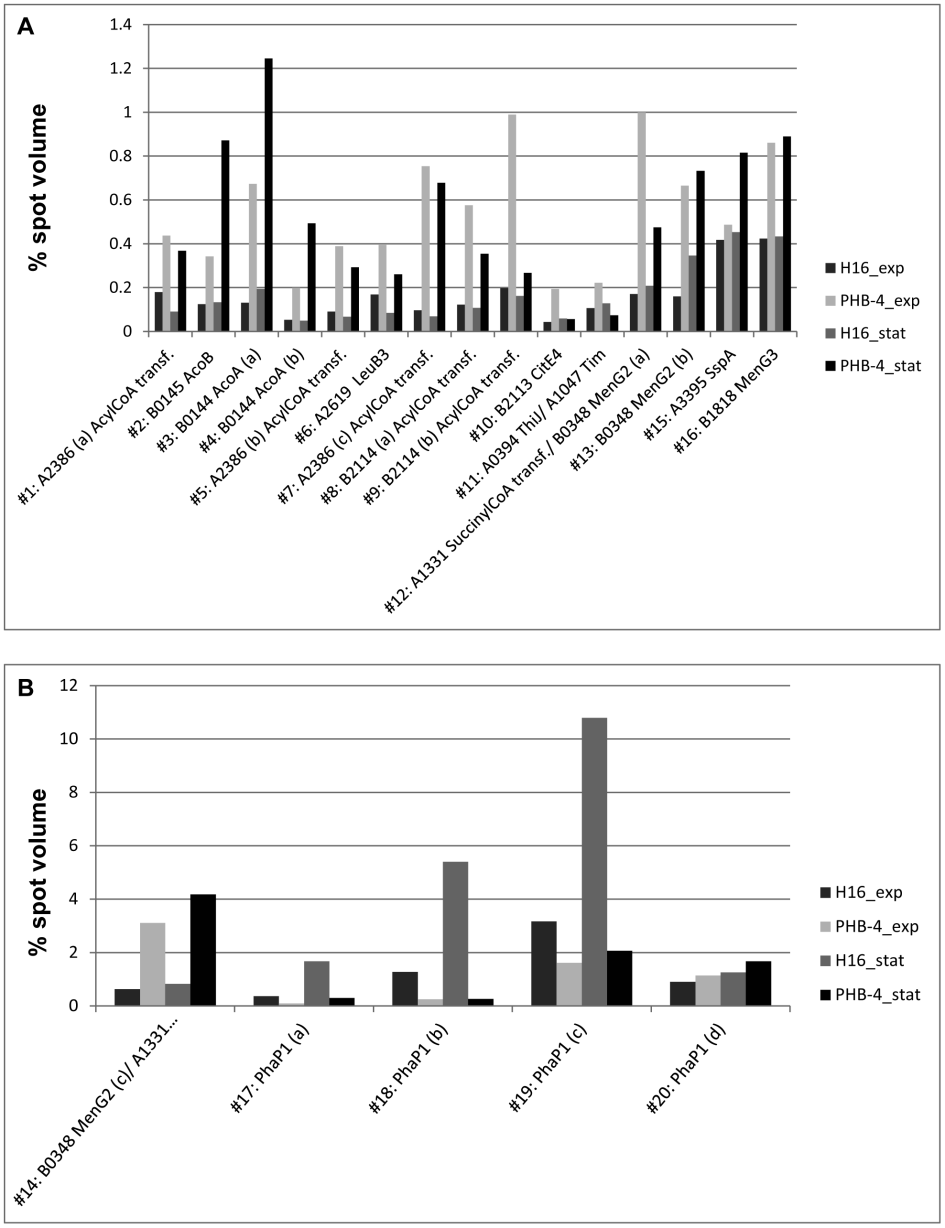
Quantification of differentially expressed proteins in *R. eutropha* H16 and mutant *R. eutropha* PHB^-^4 as based on image fusion of 2D PAGE gels (Fig. 2). Spot quantities are given as % volume (representing the relative portion of an individual spot of the total protein present on the respective average fusion image). To facilitate comparision of spot quantities, panel A presents spots with relative quantities close to 1, while spots with higher quantities are shown in panel B. Quantification was done with Delta 2D software. Suffixes (a, b, and others) indicate isoforms of the same protein species present in the gels.

Differentially expressed proteins were identified applying the DECODON Delta 2D software (Decodon GmbH, Greifswald), protein spots were excised from 2D gels and subjected to MALDI-TOF analysis. Doing so, protein species from a total of 20 spots were identified. These spots were marked and numbered in the dual views of the average fusions of the replicate 2D gel groups (Fig. 2), the corresponding proteins were listed (table 2) and densitometrically quantified according to the respective relative spot volume in the gels (Fig. 3). While 17 proteins were increasingly expressed in mutant PHB^-^4, four protein spots (#17–20) showed larger amounts in gels of the wild type H16 and consisted of isoforms of the same protein (PhaP1) with same M_w_ but differing pI's. Phasin PhaP1 represents the quantitatively predominat phasin protein of seven homologues found in *R. eutropha* H16 [53]–[55], shielding the surface of PHA granules from the cytosol [56].

**Table 2:** Differentially expressed proteins obtained by proteome analysis.

Spot #	Rank	Annotation	Accession No.
1	1	Predicted acyl-CoA transferase/carnitine dehydratase	H16_A2386
2	1	AcoB acetoin dehydrogenase E1 component beta-subunit (EC 1.2.4.1)	H16_B0145
3	1	AcoA acetoin dehydrogenase E1 component alpha-subunit (EC 1.2.4.1)	H16_B0144
4	1	AcoA acetoin dehydrogenase E1 component alpha-subunit (EC 1.2.4.1)	H16_B0144
5	1	Predicted acyl-CoA transferase/carnitine dehydratase	H16_A2386
6	1	LeuB3 3-Isopropylmalate dehydrogenase (EC 1.1.1.85)	H16_A2619
7	1	Predicted acyl-CoA transferase/carnitine dehydratase	H16_A2386
8	1	Predicted acyl-CoA transferase/carnitine dehydratase	H16_B2114
9	1	Predicted acyl-CoA transferase/carnitine dehydratase	H16_B2114
10	1	CitE4 citrate lyase beta subunit (EC 4.1.3.6)	H16_B2113
11	1	ThiJ putative intracellular protease/amidase/DJ-1/PfpI family	H16_A0394
	2	Tim triosephosphate isomerase (EC 5.3.1.1)	H16_A1047
12	1	Succinyl-CoA:3-ketoacid-coenzyme A transferase subunit A (EC 2.8.3.5)	H16_A1331
	2	MenG2 demethylmenaquinone methyltransferase (EC 2.1.1.163)	H16_B0348
13	1	MenG2 demethylmenaquinone methyltransferase (EC 2.1.1.163)	H16_B0348
14	1	MenG2 demethylmenaquinone methyltransferase (EC 2.1.1.163)	H16_B0348
	2	Succinyl-CoA:3-ketoacid-coenzyme A transferase subunit A (EC 2.8.3.5)	H16_A1331
15	1	SspA stringent starvation protein A (glutathione S-transferase) (EC 2.5.1.18)	H16_A3395
16	1	MenG3 demethylmenaquinone methyltransferase (EC 2.1.1.163)	H16_B1818
17	1	PhaP1 Phasin (PHA-granule associated protein)	H16_A1381
18	1	PhaP1 Phasin (PHA-granule associated protein)	H16_A1381
19	1	PhaP1 Phasin (PHA-granule associated protein)	H16_A1381
20	1	PhaP1 Phasin (PHA-granule associated protein)	H16_A1381

Among the proteins being increasingly expressed in strain PHB^-^4, five isoforms of an AcylCoA-transferase were observed. Expression of these protein species interestingly originated from two different gene loci, namely H16_A2386 (#1, 5, 7) and H16_B2114 (#8, 9). Generally, CoA-transferases catalyse reversible transfer reactions of coenzyme A groups from CoA-thioesters to free acids [57]. Furthermore, the α- and β-subunits of the E1 component of the acetoin dehydrogenase complex (AcoA, AcoB) showed higher synthesis in *R. eutropha* PHB^-^4 as indicated by spots 2, 3 and 4. The acetoin dehydrogenase complex (ADHC) shares a related structure with the family of 2-oxoacid dehydrogenase complexes. These multienzyme complexes consist of three principal enzyme components: a substrate-specific decarboxylase/dehydrogenase (E1), a complex-specific dihydrolipoamide acetyltransferase (E2), and a nonspecific dihydrolipoamide dehydrogenase (E3) [58], [59]. In *R. eutropha*, acetoin is metabolized by the ADHC to acetaldehyde and acetyl-CoA [60], [61]. Spot 11 consisted mainly of ThiJ and was likely stronger expressed in the mutant. ThiJ and its close orthologes are kinases involved in the biosynthesis of thiamine pyrophosphate (TPP) [62]. TPP is an essential cofactor of the E1 component of the 2-oxoacid dehydrogenase complexes [63], [64]. Additionally, the citrate lyase CitE4 (β subunit, #10) showed a strong expression, especially in the exponential growth phase of PHB^-^4. This enzyme influences the tricarboxylic acid cycle (TCC) by an anaplerotic reaction as this enzyme catalyses the conversion of citrate to acetate and oxaloactetate, and vice versa [65]. The succinyl CoA transferase (α subunit) being apparent in two spots (#12 and 14) was another protein connected to the TCC that was identified. The succinyl-CoA transferase catalyzes the transfer of coenzyme A from succinyl-CoA to acetoacetate [66]. Further proteins which showed higher expression levels were the 3-isopropylmalate dehydrogenase LeuB3 (#6), a triosephosphate isomerase (Tim, #11), the demethylmenaquinone methyltransferases MenG2 (#12–14) and MenG3 (#16) and SspA, a stringent starvation protein A (#15.)

## Discussion

### Sequence analysis of the *phaCAB* operon of the PHB-negative mutant *R. eutropha* PHB^-^4

The observed G320A point mutation introduced a stop codon in *phaC* of *R. eutropha* PHB^-^4 (Fig. 1), leading to a significantly truncated PHA synthase PhaC. Consequently, a complete loss of the catalytic activity was principally expected for the remaining PhaC protein as already shown in previous studies [20]. Supporting this assumption, Rehm presented in 2003 [67] a primary structure analysis of PhaC from *R. eutropha* summarizing the effect of various site specific mutants. These mutations were performed by Rehm and coworkers in the Steinbüchel laboratory by themselves or other laboratories and comprised insertions of *Sma*I restriction sites [68], site-specific deletions [69], and PCR-mediated random mutagenesis [70]. The corresponding data indicated that mutations in the highly variable N-terminus (the first 100 amino acid residues) or even the deletion of the entire first 100 N-terminal amino acid residues did not inactivate the enzyme. Therefore, this N-terminal region is not essential for the enzymatic activity of the PHA synthase.

In contrast, deletions at the C-terminus did abolish PHA synthase activity, suggesting that the C-terminus is essential for enzymatic activity in *R. eutropha*. Further deletions in PhaC within or between conserved blocks were not tolerated and likewise lead to an inactive enzyme, although several *Sma*I restriction side insertions between conserved blocks did not cause an inactivation of the enzyme [69]. These data together with the proposed protein model of the *R. eutropha* PHA synthase, forming a catalytic triade composed of the active site Cys-319, the conserved His-508 and the Asp-480, clearly explain the PHB-negative phenotype of *R. eutropha* PHB^-^4: the observed stop codon leads to a massively shortened and therefore inactive PHA synthase (PhaC). The results of this analysis confirm the data provided by Mifune et al. [40].

### Proteome analysis of the PHB-negative mutant *R. eutropha* PHB^-^4 in comparison to the wild type *R. eutropha* H16

Cells of *R. eutropha*, which were subjected to proteome analyses, were cultivated in MSM under conditions promoting PHB synthesis by providing a carbon source (gluconate) in excess but limiting the nitrogen source. When cells became stationary due to nitrogen limitation, cells of *R. eutropha* PHB^-^4 were faced with an excess of intermediates and products of the KDPG pathway wich metabolizes the gluconate leading to pyruvate. Part of the pyruvate is subsequently converted to acetyl-CoA by the pyruvate dehydrogenase complex (PDHC). In contrast to the wild type H16, mutant PHB^-^4 is not able to direct these amounts of acetyl-CoA towards synthesis and accumulation of PHB. Under these conditions, mutant PHB^-^4 excretes large amounts of pyruvate. This behavior is a general characteristic of all PHB-negative mutants investigated so far; pyruvate excretion was observed when spontaneous and chemically or transposon-induced mutants were exposed to conditions which are permissive for the accumulation of PHB in the wild type cells [71]–[73]. At comparable cultivation conditions to those in this study, i.e. applying MSM with exess gluconate but limited ammonium provision, the PHB-negative mutant excreted up to 40 mM pyruvate in the stationary growth phase leading to a decrease of the pH value in the medium to pH 5 [73].

**Figure 4:**
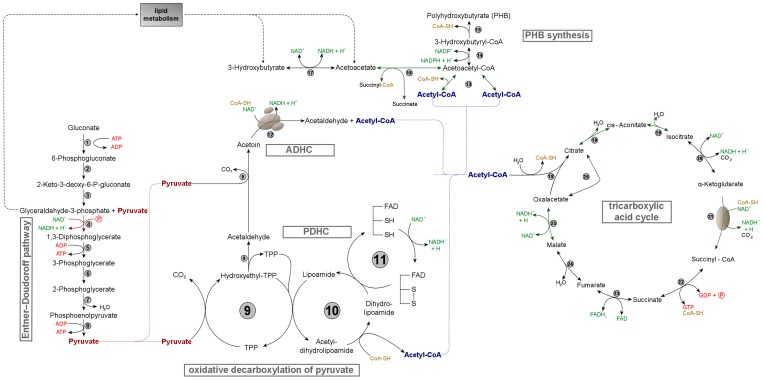
Central metabolism of *R. eutropha* H16 and mutant PHB^-^4 with regard to the results of proteome analyses. The numbers in the scheme indicate the following involved enzymes: 1, glucokinase; 2, phosphogluconate dehydratase; 3, phospho-2-keto-3-desoxygluconate aldolase; 4, glyceraldehyde-3-phosphate dehydrogenase; 5, phosphoglycerate dehydrogenase; 6, phosphoglyceromutase; 7, enolase; 8, pyruvate kinase; 9, pyruvate dehydrogenase/decarboxylase (E1 of PDHC); 10, dihydrolipoamide acetyltransferase (E1 of PDHC); 11, dihydrolipoamide dehydrogenase (E3 of PDHC); 12, acetoin dehydrogenase enzyme system; 13, acetyl-CoA acetyltransferase; 14, acetoacetyl-CoA reductase; 15, PHB synthase; 16, 3-oxoacid-CoA transferase; 17, 3-hydroxybutyrate dehydrogenase; 18, citrate synthase; 19, aconitase; 20, isocitrate dehydrogenase; 21, 2-oxoacid dehydrogenase multienzyme complex; 22, succinyl-CoA synthetase; 23, succinate dehydrogenase; 24, fumarase; 25, malate dehydrogenase; 26, citrate lyase.

The proteome analysis revealed several metabolic adaptations of mutant PHB^-^4 to this situation. Figure 4 gives a schematic overview of the relevant parts of the metabolism of *R. eutropha* H16 and mutant PHB^-^4. One protein, which was synthesized in larger amounts in the mutant, was the kinase ThiJ, which is involved in biosynthesis of TPP, the essential cofactor of the 2-oxoacid dehydrogenase complexes [63], [64]. 2-Oxo-acid dehydrogenase complexes convert 2-oxo acids to the corresponding acyl-CoA derivatives and produce NADH and CO_2_ in an irreversible reaction. Five members of this family are known at present, the above mentioned pyruvate dehydrogenase complex (PDHC), the 2-oxoglutarate dehydrogenase complex (OGDHC), the branched-chain dehydrogenase complex (BCDHC), the glycine dehydrogenase complex (GDHC) and the acetoin dehydrogenase complex (ADHC) [74]. Regarding strain PHB^-^4, the flux of pyruvate from the KDPG pathway via PDHC to acetyl-CoA and finally to PHB is impaired, obviously leading to higher celluar amounts of acetyl-CoA and pyruvate that cannot be further converted. The observed overproduction of ThiJ might indicate a strengthening of the PDHC by providing greater amounts of cofactor TPP as an answer to an excess of pyruvate. It has been indirectly shown that PDHC plays the essential role in this context, as evidence has been provided that the pyruvate dehydrogenase, which harbours the cofactor TPP, can act as a bottleneck in the conversion of pyruvate. Thiamine auxotrophic mutants of *Acinetobacter* sp. as well as some other bacteria, which suffered from thiamine deficiency, excreted pyruvate to the medium in contrast to the wild types [75]. However, it is known that the PDHC is allosterically regulated with pyruvate acting as a positive effector and acetyl-CoA acting as a negative effector [74] which might counteract the metabolization of pyruvate by PDHC, if the acetyl-CoA concentration increases.

On the other hand, α- and β-subunits (AcoA, AcoB) of the E1 component of the acetoin dehydrogenase complex (ADHC) showed also higher synthesis in *R. eutropha* PHB^-^4. The ADHC shares as a member of the family of 2-oxoacid dehydrogenase complexes a related structure and also needs TPP as cofactor. It was shown that acetoin can be formed by a side reaction of the pyruvate decarboxylase (PdhA) of the PDHC (E1 component). In a first step, the conversion of hydroxyethyl-TPP to acetaldehyde with concomitant recovery of TPP occurs [76]. Furthermore, the PdhA is able to transfer the hydroxyethyl moiety of hydroxyethyl-TPP to acetaldehyde to finally form acetoin. Then, acetoin is metabolized by ADHC to acetaldehyde and acetyl-CoA [60], [61]. This bypass might additionally counteract an accumulation of pyruvate which as an acid impairs the cell milieu, by providing an alternative path to acetyl-CoA, if PDHC was overloaded by abundant pyruvate and negatively affected by high concentrations of acetyl-CoA. Additionally, the interim accumulation of a certain amount of acetoin might be of particular advantage for the cells, as an overacidification of the intracellular environment and culture medium due to acidic product accumulation is prevented by conversion of pyruvate to uncharged acetoin [77].

Interestingly, our laboratory observed a similar effect on high cellular pyruvate concentrations in the context of a previous analysis of mutants of *R. eutropha*, which were lacking the dihydroliponamid dehydrogenase (PdhL), which constitutes the E3 component of the PDHC [78]. As the PDHC was in this case obviously not able to restore full activity by expression of homologues genes to *pdhL* encoded in *Ralstonia*'s genome, the cells likewise generated acetyl-CoA from pyruvate via ADHC and excreted excessive pyruvate.

The resulting acetyl-CoA subsequently enters the tricarboxylic acid cycle (TCC): the citrate synthase catalyzes the condensation reaction of acetyl-CoA and oxaloacetate to form citrate, which is further oxidized in the TCC. Oxaloacetate will be regenerated after the completion of one round of the TCC, or can be additionally provided by anaplerotic reactions [79]. The citrate lyase CitE4, which was found to be increasingly expressed in proteome analysis, catalyzes the reaction of citrate to acetate and oxaloacetate [65]. This reaction can obviously directly regenerate oxaloacetate and could therefore provide a direct anaplerotic link from citrate to oxaloacetate thereby circumventing the consecutive reaction steps of the TCC. Larger quantities of oxaloacetate are probably needed for the conversion of the enhanced amounts of acetyl-CoA which can not be directed towards PHB synthesis in mutant PHB^-^4. Indeed, experiments of Ruhr [80] showed a 2.2-fold higher intracellular concentration of acetyl-CoA in autotrophic cells of mutant PHB^-^4 when ammonium was absent. The corresponding expected lower intracellular concentration of free CoA in turn negatively influences the catalytic rate of the pyruvate dehydrogenase and supports excretion of pyruvate [73].

The upregulated kinase ThiJ may likewise enhance the activity of the OGDHC (or alternatively α-ketoglutarate dehydrogenase complex), which in a later step decarboxylates α-ketoglutarate to succinyl-CoA with concomittant reduction of NAD^+^, since TPP acts also as cofactor in this multienzyme complex. Succinyl-CoA is subsequently converted so succinate by means of the succinyl-CoA synthetase under generation of GTP. Interestingly, a succinyl CoA-transferase (H16_A1331), which catalyzes the transfer of coenzyme A from succinyl-CoA to acetoacetate or vice versa [66], showed much stronger expression in strain PHB^-^4. This indicates that this transferase may play a role in transferring CoA from acetoacetyl-CoA to succinate in order to reduce the acetoacetyl-CoA pool, because the latter will accumulate in mutant PHB^-^4 as a direct precursor of PHB due to the lack of an active PHA synthase in this strain.

Furthermore, five isoforms of two different acyl-CoA transferases (H16_A2386, H16_B2114) were observed among the upregulated proteins. Generally, CoA-transferases catalyse reversible transfer reactions of coenzyme A from CoA-thioesters to free acids [57] and can exhibit relative broad substrate specificities [81]. The precise donor and acceptor molecules of these two acyl-CoA transferases of *R. eutropha* are unknown, yet. However, the significant increased synthesis of acyl-CoA transferases in mutant PHB^-^4 indicates a need for transfer reactions between organic acid intermediates as a response of impaired PHB accumulation.

Two demethylmenaquinone methyltransferases, MenG2 and MenG3, which catalyze the carbon methylation reaction during biosynthesis of ubiquinone (coenzyme Q) and menaquinone (vitamin K2), were significantly stronger synthesized in strain PHB^-^4. Both isoprenoid quinones are essential components of the respiratory electron transport chain [82]. Apparently, the capacity of the respiratory electron transport chain is improved to address the high amounts of reduction equivalents from TCC in PHB^-^4 and to accept electrons from NADH and succinate.

Another protein, which was synthesized in larger amounts in the mutant, was 3-isopropylmalate dehydrogenase, LeuB3. This enzyme is involved in the biosynthesis of valine, leucine and isoleucine from pyruvate [83]. Enhanced concentrations of this enzyme may additionally help to reduce the pyruvate pool. The glutathione transferase SspA showed very high amounts in cells of strain PHB^-^4 in the stationary growth phase. Bacterial glutathione transferases (GSTs) are part of a superfamily of enzymes that play a key role in cellular detoxification. GSTs are widely distributed in prokaryotes and are grouped into several classes. Bacterial GSTs are implicated in a variety of distinct processes such as the protection against chemical and oxidative stresses [84]. Higher amounts of SspA may address the stress obviously induced by the loss of directing metabolic intermediates and reduction power to PHB synthesis in mutant PHB^-^4.

Four isoforms of the predominant phasin PhaP1 shielding the surface of PHA granules from the cytosol [56] were found to be massively stronger synthesized in the wild type H16 in comparison to the PHB-negative mutant PHB^-^4, especially in the stationary growth phase where PHB synthesis reaches its maximum. Increased expression is strictly associated with the accumulation of PHB, when phasins constitute the major component of the boundary layer at the surfaces of PHB granules [53] and has been observed in other proteome analyses before [48].

In summary, the proteomic changes of mutant PHB^-^4, which has lost the ability to synthesize and accumulate PHB, provide a deep insight into the cells' metabolic situation: The cells are obviously faced with high concentrations of accumulating intermediates such as pyruvate and acetyl-CoA [73]. Mutant PHB^-^4 tries to prevent acidification by (a) excretion of pyruvate and by (b) stronger synthesis of the observed protein species: Increased expression of (i) ThiJ supports biosynthesis of cofactor TPP and thereby reinforces the 2-oxoacid dehydrogenase complexes PDHC, ADHC and OGDHC in order to convert pyruvate at a higher rate. Additionally, the (ii) 3-isopropylmalate dehydrogenase LeuB3 may direct pyruvate into synthesis of different amino acids. Larger amounts of acetyl-CoA that cannot be channeled to PHB synthesis in mutant PHB^-^4 are countered by increased synthesis of (iii) different acylCoA-transferases which allow transfer reactions between organic acid intermediates. A large fraction of acetyl-CoA enters the TCC, and (iv) the increasingly expressed citrate lyase CitE4 can regenerate oxaloacetate from citrate for the conversion with acetyl-CoA in a kind of direct anaplerotic link. Substantial amounts of reduction equivalents that emerge inTCC are countered by (v) enhancing capacity of the respiratory chain through synthesis of more ubiquinones as indicated by the observed stronger synthesis of MenG2 and MenG3. Finally, the (vi) stronger expressed glutathione transferase SspA could reduce oxidative stress caused by the loss of PHB synthesis in mutant PHB^-^4.
